# The transcriptome of zinc deficient maize roots and its relationship to DNA methylation loss

**DOI:** 10.1186/s12870-018-1603-z

**Published:** 2018-12-27

**Authors:** Svenja Mager, Brigitte Schönberger, Uwe Ludewig

**Affiliations:** Institute of Crop Science, Nutritional Crop Physiology, University of Hohenheim, Fruwirthstr. 20, 70593 Stuttgart, Germany

**Keywords:** Zinc, DNA methylation, Maize, Gene expression, Genome stability, Transposable elements

## Abstract

**Background:**

Zinc (Zn) is an essential micronutrient of all organisms. Deficiency of zinc causes disturbance in crucial plant functions, as a high number of enzymes, including transcription factors, depend on zinc for proper performance. The plant responses to zinc deficiency are associated with increased high affinity Zn uptake and translocation, as well as efficient usage of the remaining zinc, but have not been characterized in molecular detail in maize.

**Results:**

The high affinity transporter genes *ZmZIP3,4,5,7* and *8* and nicotianamine synthases, primarily *ZmNAS5*, were identified as primary up-regulated in maize roots upon prolonged Zn deficiency. In addition to down-regulation of genes encoding enzymes involved in pathways regulating reactive oxygen species and cell wall-related genes, a massive up-regulation of the sucrose efflux channel genes *SWEET13a,c* was identified, despite that in –Zn sugar is known to accumulate in shoots. In addition, enzymes involved in DNA maintenance methylation tended to be repressed, which coincided with massively reduced DNA methylation in Zn-deficient roots. Reduced representation bisulfate sequencing, which revealed base-specific methylation patterns in ~ 14% of the maize genome, identified a major methylation loss in -Zn, mostly in transposable elements. However, hypermethylated genome regions in –Zn were also identified, especially in both symmetrical cytosine contexts. Differential methylation was partially associated with differentially expressed genes, their promoters, or transposons close to regulated genes. However, hypomethylation was associated with about equal number of up- or down-regulated genes, questioning a simple mechanistic relationship to gene expression.

**Conclusions:**

The transcriptome of Zn-deficient roots identified genes and pathways to cope with the deficiency and a major down-regulation of reactive oxygen metabolism. Interestingly, a nutrient-specific loss of DNA methylation, partially related to gene expression in a context-specific manner, may play a role in long-term stress adaptation.

**Electronic supplementary material:**

The online version of this article (10.1186/s12870-018-1603-z) contains supplementary material, which is available to authorized users.

## Background

Zinc (Zn) is an essential micronutrient needed by every organism on earth. It is an important cofactor of a high number of transcription factors and enzymes in plants [1]. Zinc can function as structural component aiding in appropriate protein folding and as a catalytic component, enabling or enhancing the reactions performed by enzymes. Additionally, zinc is also needed for proper membrane integrity and takes part in RNA and DNA metabolism, as well as gene expression regulation. It is further involved in detoxification of superoxide radicals and synthesis of phytohormones [1–4]. Considering these many functions of zinc in plants, it is not surprising that plants suffer strongly from prolonged zinc deficiency (-Zn) and have developed a range of coping mechanisms. Zn deficiency is especially widespread in areas of calcareous, sandy or peat soils. Due to their high pH, which causes Zn adsorption to clay or calcium carbonate, the bioavailable Zn that can be absorbed by the roots remains limited, despite substantial Zn content in such soils. In waterlogged soils, effecting world-wide flooded rice production, the risk for Zn deficiency is heightened due to zinc forming sparingly soluble compounds in the oxidized rhizosphere [5]. All in all, it is estimated that about 50% of cereal crop agricultural soils are potentially zinc deficient [5].

In maize, Zn deficiency leads to stunted growth and morphological abnormalities, such as lower vascular bundle proportion and higher stomatal density, among changes in physiology, such as decreased net photosynthesis and lowered stomatal conductance [6]. It is well-established that under Zn deficiency, zinc transporter genes from the ZIP (Zrt/Irt-like proteins) family are up-regulated to facilitate the uptake of traces of remaining zinc [2, 7, 8], but in maize, these genes were only marginally up-regulated in -Zn in shoots and roots [9]. Furthermore, in a Zn tolerant genotype, Zn transporter genes *ZmZIP1, ZmZIP4* and *ZmIRT1* were moderately up-regulated by Zn deficiency, but not in a sensitive genotype [10].

But not only plants need a sufficient amount of zinc, also animals and humans are negatively affected by zinc deficiency. Among humans, especially widespread in developing countries, zinc deficiency concerns over 2 billion people. Zinc in humans is needed by the immune system, for DNA synthesis and RNA transcription, as well as in cell division and prevention of apoptosis [11]. Health consequences of zinc deficiency range from weight loss over emotional disorder to impairment of the immune system, to mention only some [12]. In respect to that, sufficient intake of zinc via crop products helps to alleviate this nutritional disorder. One approach for achieving this is biofortification, to increase the nutritional value of important crop plants by breeding. Knowledge about plant mechanisms for using zinc or coping with zinc deficiency is imperative. Substantial knowledge on how plants handle zinc is available, although molecular mechanisms of zinc homeostasis in plants are still fragmentary [2]. More recently, DNA methylation changes were reported for many stresses, including nutrient stresses. In various cases, for example phosphorus starvation, gene expression changes were correlated with DNA methylation differences, especially in the dynamic, non-symmetrical cytosine context [13, 14]. This methylation context, also responsible for *de-novo* DNA methylation, is dependent on the RNA-directed DNA methylation (RdDM) pathway, in which sRNAs play an important role. In this pathway, plant-specific DNA-dependent RNA polymerases produce short RNA transcripts, from which double-stranded RNAs (dsRNAs) are produced by an RNA-dependent RNA polymerase. In turn, these dsRNAs are then cleaved by a protein called dicer-like 3 (DCL3) to form 21 to 24 nucleotide sRNAs, which are then incorporated into argonaute 4 (AGO4). This complex then guides DNA methylation with sequence information from the RNAs [15–17].

We hypothesized that major gene categories that have been identified as regulated under Zn deficiency in model plants are also similarly regulated in Zn-deficient maize roots. Maize-specific genes, however, might also be identified. We therefore performed RNA-sequencing on well-supplied and zinc-deficient maize roots. While typical Zn deficiency response genes were identified, we noted that genes involved in DNA methylation were also affected by -Zn and therefore quantified the impact of Zn deficiency on the DNA methylome. DNA methylation in plants is suggested to influence different plant functions, as for example aiding in the pathogen response, genome stability, heterosis, as well as suppression of transposable elements (TEs) and genes [18, 19]. Whether and how DNA methylation is correlated to gene expression under Zn deficiency is presented and discussed.

## Results

### Phenotype and nutrient concentrations of maize plants

**Fig. 1:**
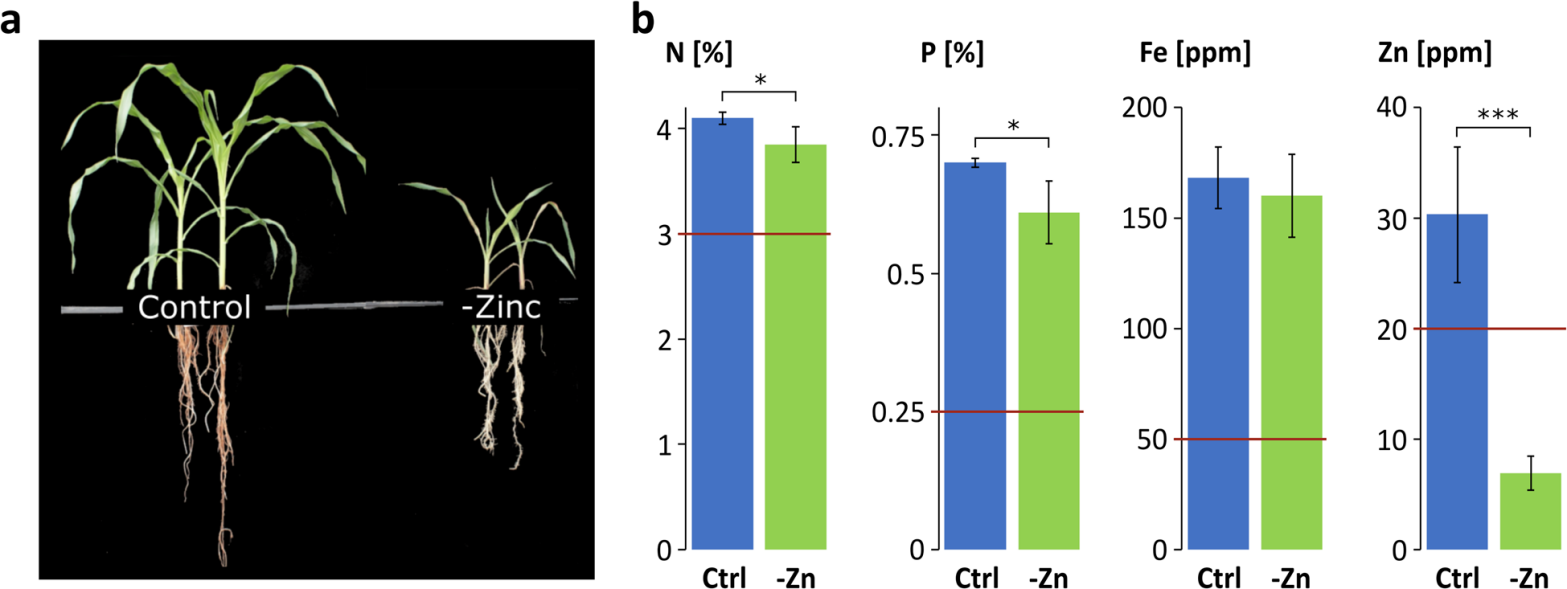
Plant phenotypes and shoot nutrient concentrations (**a**) Phenotype of control and -Zn plants at harvest. **b** Concentrations of N, P, Fe and Zn in control (blue bars) and -Zn (green bars) shoots (*n* = 3). The red lines indicate sufficiency levels for maize

Phenotypic investigation of control and -Zn plants showed vigorous growth of control plants, while -Zn plants showed typical Zn deficiency-induced symptoms with strongly stunted growth. The leaf and root size were reduced and chlorotic and necrotic marks on the leaves were visible [3] (Fig. 1a). Nutrient analysis confirmed the Zn deficiency in shoots of -Zn plants, whereas iron, which can potentiate reactive oxygen stress and is frequently higher in field conditions under Zn deficiency, was close to control level (Fig. 1b). Zn was at only 6.9 ppm, while control plants contained more than 30 ppm Zn and sufficient amounts of all other tested nutrients, although the Zn-deficient plants had slightly reduced nitrogen and phosphorus levels (Fig. 1b).

### Zinc-deficiency adapted transcriptome

**Fig. 2:**
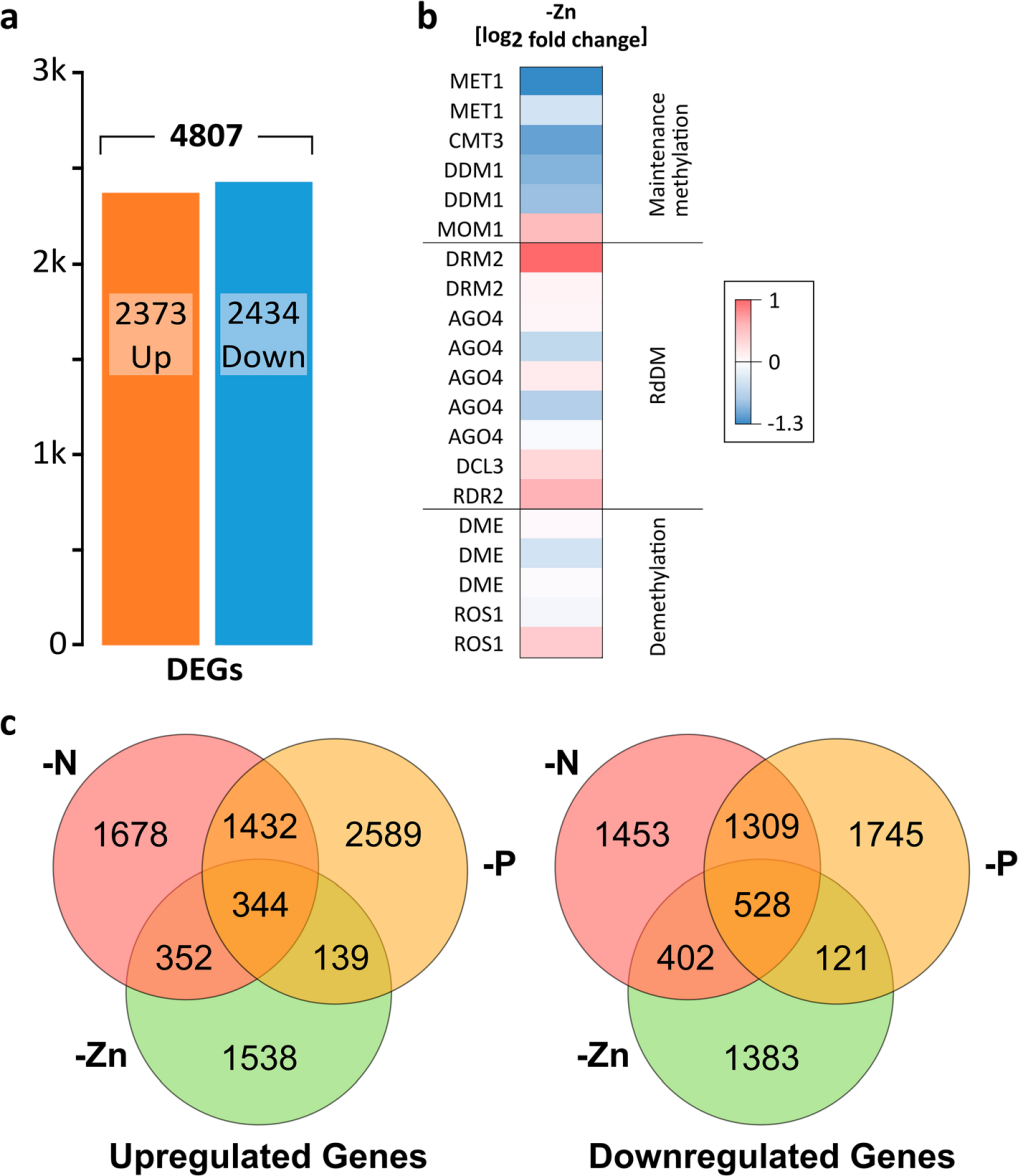
Differential gene expression in -Zn **(a)** Number of differentially expressed genes (DEGs, *p* < 0.05), either up- (orange) or down-regulated (blue) in Zn deficiency. **b** Log_2_-fold differences in gene expression of putative genes involved in DNA methylation and demethylation. Blue: down-regulated, red: up-regulated. **(c)** Overlap of up-regulated (left) and down-regulated (right) genes in different deficiencies (-Zn, -N, -P); nitrogen and phosphorus deficiency data from [22]

Root transcriptomes under prolonged Zn deficiency were then obtained and compared to controls. Of the RNA samples taken from control and -Zn roots, the alignment rate was higher in controls (91.8%) compared to 79.6% in -Zn (Additional file 1:Table S1). The aligned transcripts were assembled and analyzed for differentially expressed genes. Altogether, 4807 significantly differentially expressed genes (DEGs) were identified between the control and the -Zn treatment. An almost equal number of genes were up- or down-regulated, namely 2372 and 2434, respectively (Fig. 2a). In contrast to short or mild Zn deficiencies tested previously [9], several genes coding for zinc uptake systems, *ZmZIP3, ZmZIP4, ZmZIP5, ZmZIP7* and *ZmZIP8,* were substantially up-regulated (Table 1). Additionally, genes encoding for nicotianamine synthase (NAS), especially the abundant *ZmNAS5*, were consistently up-regulated during zinc deficiency. NAS synthesizes nicotianamine, which in turn is involved in translocation of heavy metals, as for example zinc and iron, between organs. Zinc is needed for detoxification of superoxide radicals via the copper/zinc-dependent superoxide dismutase and the transcripts of this enzyme were down-regulated under zinc deficiency, consistent with results from many other plants [3]. Strong down-regulation was also discovered in many peroxidases, where 55 out of the 63 differentially expressed peroxidases were reduced in their expression level (two of them are exemplary shown in Table 1). Carbonic anhydrase, well-known to require Zn as a co-factor and catalyzing CO_2_ hydration, was also reduced in -Zn (Table 1). There was an increase in purple acid phosphatases, which help maintaining inorganic phosphate metabolism and may be used to mobilize Zn that is in complex with phosphate [20]. Expansins, by contrast, which play roles in plant cell expansion, were down-regulated under -Zn, in agreement with smaller organ sizes in -Zn. Interestingly, a strong up-regulation of the sugar efflux transporter genes *SWEET13a* and *c*, which are involved in phloem loading of sucrose in the leaves [21], was noted (Table 1).

**Table 1:** Examples of differentially expressed genes under Zn deficiency

Gene ID	Annotation	FPKM Control	FPKM -Zn	Log_2_ FC
GRMZM2G111300	ZIP-type metal cation transporter ZmZIP4	68.3	255.7	1.90
GRMZM2G045849	ZIP-type metal cation transporter ZmZIP3	52.9	106.0	1.02
GRMZM2G064382	ZIP-type metal cation transporter ZmZIP5	8.3	154.6	4.21
GRMZM2G015955	ZIP-type metal cation transporter ZmZIP7	114.9	195.9	0.77
GRMZM2G047762	ZIP-type metal cation transporter ZmZIP1-related	26.2	134.9	2.37
GRMZM2G093276	ZIP-type metal cation transporter ZmZIP8	20.6	119.6	2.54
GRMZM2G106928	Superoxide dismutase	58.0	25.6	−1.18
GRMZM2G175728	Superoxide dismutase	11.7	3.2	−1.89
GRMZM2G410175	Peroxidase	66.6	0.9	−6.3
GRMZM2G048474	Peroxidase	76.1	3.5	−4.4
GRMZM2G478568	Nicotianamine synthase ZmNAS3	19.2	45.1	1.23
GRMZM2G050108	Nicotianamine synthase ZmNAS5	35.5	186.7	2.40
GRMZM2G312481	Nicotianamine synthase ZmNAS8	0.4	2.2	2.64
GRMZM2G385200	Nicotianamine synthase ZmNAS1	1.5	8.5	2.49
GRMZM2G034956	Nicotianamine synthase ZmNAS10	5.0	0.4	−3.60
GRMZM2G348512	Carbonic Anhydrase 2	230.8	125.3	−0.88
GRMZM2G046924	Carbonic Anhydrase 1	89.2	54.1	−0.72
GRMZM2G073860	Purple acid phosphatase 10	7.8	17.9	1.19
GRMZM2G134054	Purple acid phosphatase 15	3.90	10.9	1.48
GRMZM2G133322	sugar efflux transporter ZmSWEET12a	1.2	78.3	6.07
GRMZM2G060974	sugar efflux transporter ZmSWEET3b	17.5	0.3	−5.77
GRMZM2G173669	sugar efflux transporter ZmSWEET13a	75.2	1229.6	4.03
GRMZM2G179349	sugar efflux transporter ZmSWEET13c	7	79.2	3.51
GRMZM2G139834	Nodulin MtN21 family protein	29.1	2.6	−3.51
GRMZM2G467893	Nodulin family protein	11.1	128.7	3.54
GRMZM2G001035	vacuolar iron transport-like	70.3	1.7	−5.34
GRMZM2G173826	Expansin-A12 precursor EXP11	15.4	1.7	−3.16
GRMZM2G450546	Expansin-A19 precursor EXP11	6.3	0.3	−4.51
GRMZM2G021427	Expansin-B3 precursor EXPB2	20.6	2.3	−3.15
GRMZM2G327266	Expansin-B11 precursor	39.8	4	−3.31
	EXPB4			

*FPKM* Fragments per kilobase of transcript per million mapped reads, *Log*_*2*_ *FC* log_2_(FPKM -Zn/FPKM Control)

Gene enrichment analysis revealed a strong overrepresentation of down-regulated categories with genes coding for enzymes that are involved in the oxidative stress response, like the before mentioned peroxidases and superoxide dismutases. The three most significant down-regulated GO term categories contained genes involved in the oxidative stress response (Table 2). Despite that the total number of individual up- and down-regulated genes was similar, the down-regulated gene categories were much more pronounced than significantly up-regulated categories, which only comprised a single GO term, which was related to production of secondary metabolites.

**Table 2:**
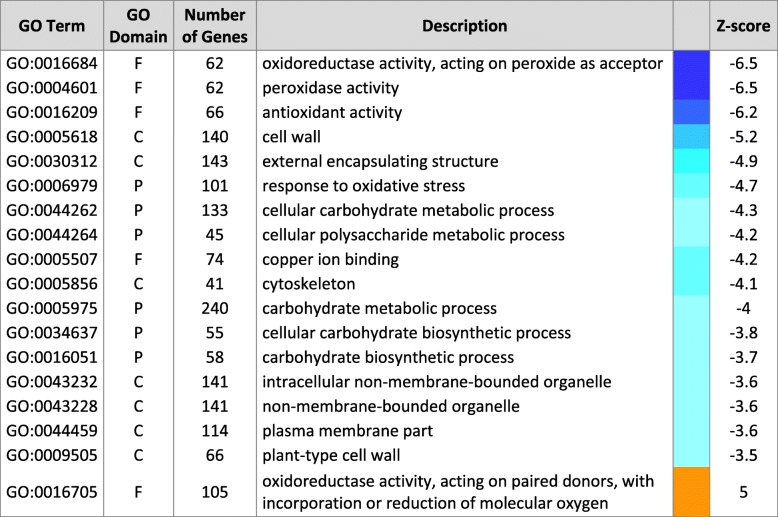
Over-representation of gene ontology (GO) categories in -Zn roots

Bonferroni multi-test adjusted Parametric Analysis of Gene Set Enrichment (PAGE) of log_2_ fold-change of DEGs. GO domain: F, molecular function; C, cellular compartment; P, biological process. Top 18 categories are shown. Color code: Blue = underrepresentation, orange = overrepresentation of gene category in -Zn

Interestingly, several genes coding for maintenance methylation enzymes also tended to be down-regulated, while some demethylating enzymes were up-regulated (Fig. 2b). Genes involved in the RdDM pathway were less consistently regulated, but it is important to note that the expression of (de-)methylation-related genes was altogether relatively low, somewhat questioning the significance of the observed differences.

When comparing differential expression under zinc deficiency with previously analyzed differential expression under nitrogen or phosphorus deficiency [22], we noted a small overlap of genes that were up- or down-regulated under all three deficiencies (344 and 528, respectively, Fig. 2c). A much larger number of genes, however, was specifically associated with individual nutrient deficiencies and these included the genes associated with -Zn discussed above.

### Adaptation of DNA methylation to Zn deficiency

The transcriptional data suggested that -Zn adapted roots might be demethylated, which was experimentally tested by reduced representation bisulfite sequencing of DNA extracted from the same root material, from which nutrient analyses and RNA-seq profiles were generated. These data provided sequence-specific methylation information for about 14% of the maize genome (~ 18% of all cytosines) in each sample. The conversion rate after bisulfite treatment for each sample was > 98% (between 98.41 and 99.18%). About 90% of all cytosines were covered by at least 5 reads in all contexts (Additional file 1: Figure S1) and mappability was > 48% (Additional file 1: Table S2) indicating sufficient coverage and mappability for reliable downstream analyses.

**Fig. 3:**
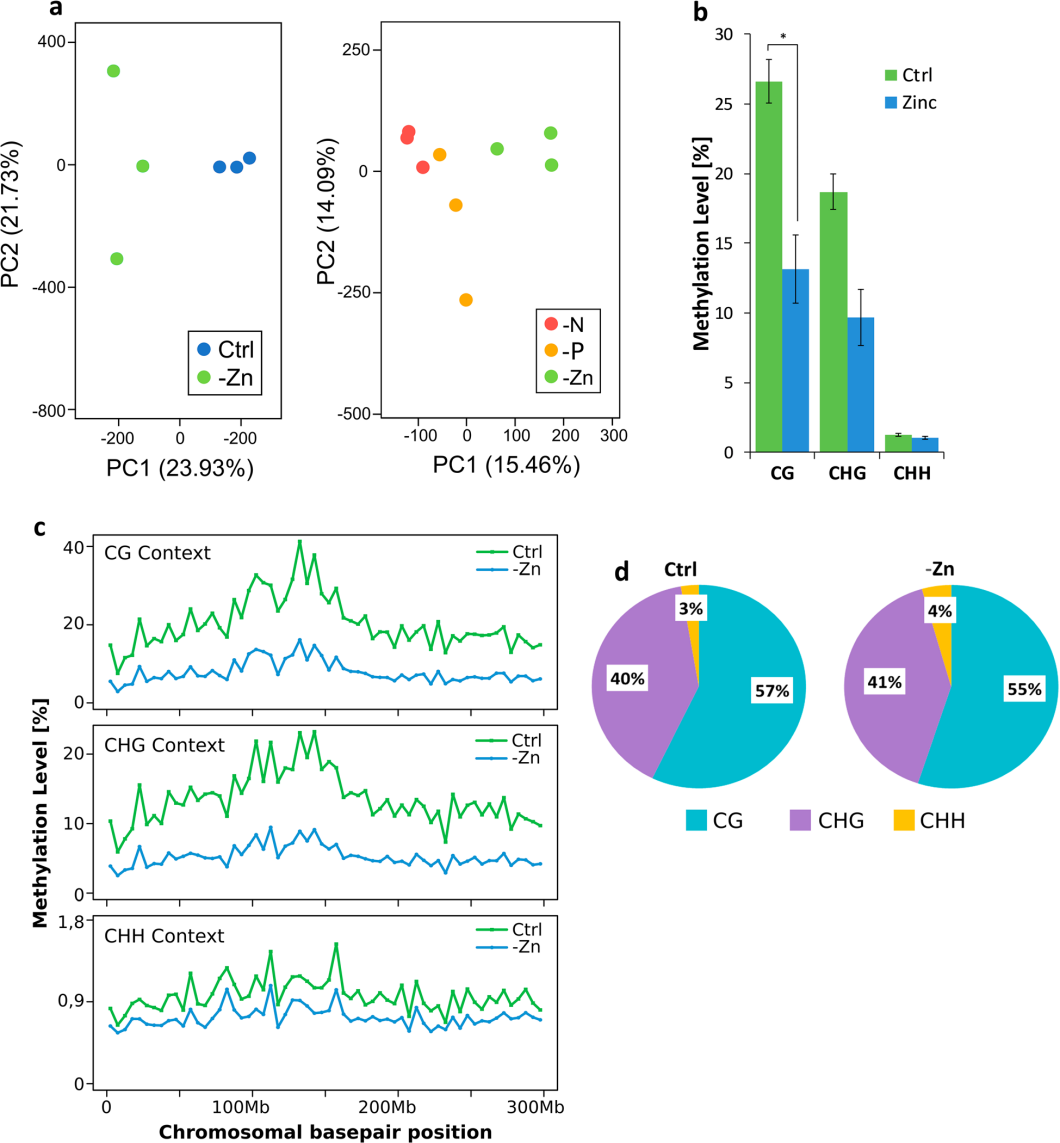
DNA methylation level **(a)** Principle component analysis (PCA) of reduced representation bisulfite sequencing cytosine methylation data. Left diagram: control condition (blue) and -Zn (green). Right diagram: zinc, nitrogen and phosphorus deficiencies, data partially from [22]. The first two PCs are shown. **b** Overall methylation level (in percent) in all contexts, control: blue; -Zn: green. **c** Average (across 5 million basepair grids) methylation level in chromosome 1 in all contexts. **d** Contribution of each context methylation to the total methylation level, control: left; -Zn: right

Principal component analysis was performed on the methylation levels of the cytosines of control and -Zn samples (Figure 3a). The variance of principle component 1 (PC1) explained about 24% of the total variance and separated the control and -Zn samples into two groups. PC2 was apparently mainly due to variances between the replicates of -Zn. We also compared our dataset to data from -N and -P treated maize roots [22], which revealed that the methylome samples for each nutrient deficiency were separated mainly by PC1. This variance explained about 15% of the total variance between the three treatments.

A massive loss of methylation in the CG and CHG contexts (H = A, T or C) in -Zn was found (Fig. 3b). In the control, 26.6% of all cytosines in the CG context were methylated, whereas in -Zn samples, only about half as many CGs were methylated (13.2%). There was also a significant loss of methylation for -Zn samples in the CHG context, where 9.7% were methylated in -Zn, compared to 18.7% in control samples. The CHH context was almost unaffected by the deficiency. Cytosines in this context showed only very low methylation, as already found in our previous analysis [22]. This methylation level was slightly further reduced in -Zn samples, from 1.26% in control to 1.06% in -Zn.

The methylation distribution across whole chromosomes was analyzed by DMRcaller (Fig. 3c). The methylation level in the three different cytosine contexts in chromosome 1 is shown as a representative example. Especially in the CG and CHG context, the profiles show that the methylation was higher at the centromeric region and decreased towards the ends of the chromosomes. Again, the CHH context formed an exception, as here the higher methylation at the centromere was almost not visible. The reduction of CG and CHG methylation was relatively uniform, though most methylation seemed to be lost at centromeric and pericentromeric regions, reducing the comparably higher overall methylation level in centromeric regions. In all chromosomes, the reduced methylation in -Zn was less pronounced in the CHH context than in the other two contexts.

Even though a lot of methylation was lost in the deficiency samples, a similar fraction of the total number of methylated cytosines was found in each context (Fig. 3d). This showed that the same relative amount of methylation is lost in each context.

Individual base methylation changes are currently thought to have little functional relevance, so only differentially methylated regions (DMRs) were considered. We defined DMRs as regions between 50 and 500 bp that contain at least 4 cytosines, more than 3 reads per cytosine were required, which differed in methylation by more than 40% and a p-cutoff value of ≤0.01. 2762 DMRs were identified in total, with most DMRs in the CG context (Table 3). In the CHG context, 402 DMRs were identified. By contrast, in the CHH context only 3 DMRs were found. With less strict cutoff values for DMRs (minimum of 3 cytosines, at least 3 reads per cytosine and at least 10% methylation difference) 41 DMRs were identified (Table 3).

**Table 3:** Number of DMRs resulting from strict and loosened criteria defining a DMR

	Strict Criteria	Loose Criteria
CG	2762	10,036
CHG	402	2897
CHH	3	41

**Fig. 4:**
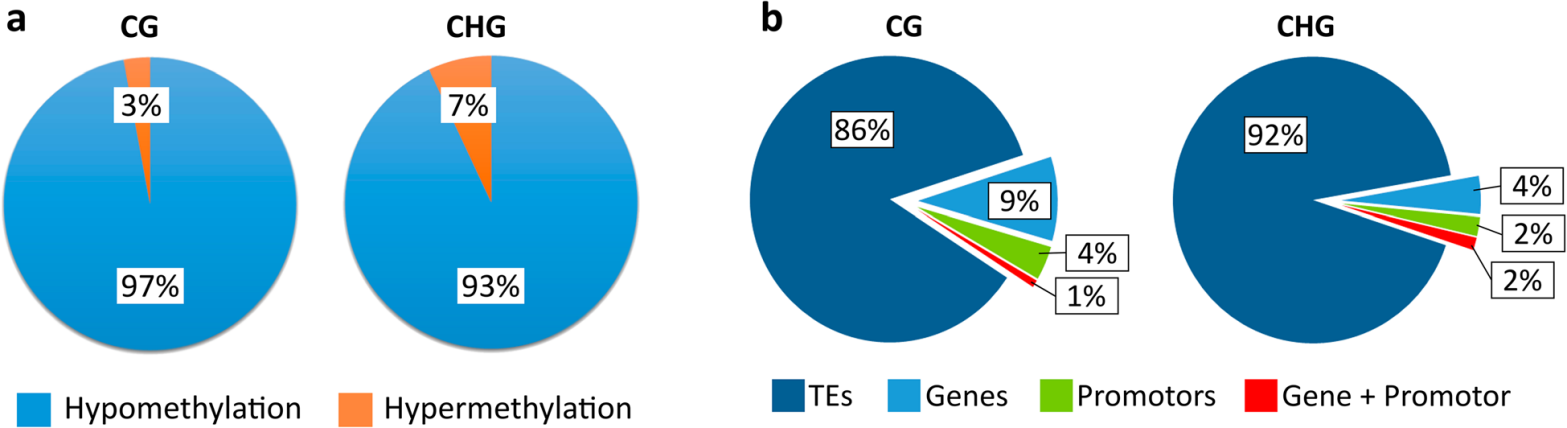
Methylation change and location of DMRs **(a)** Percentage of DMRs being hypo- (blue) or hypermethylated (orange) in CG context (left) and CHG context (right). **b** Percentage of DMRs located in transposable elements (dark blue), genes (blue), promoters (green) or spanning promoter and gene (red) in CG context (left) and CHG context (right)

As in -Zn the overall methylation was massively decreased in the CG and CHG contexts, DMRs were preferentially hypomethylated, but hypermethylated regions in –Zn were also identified. This indicated that in addition to the methylation loss, further de novo methylation in -Zn occurred. In total, about 93–98% of all DMRs were hypomethylated (Fig. 4a).

DMRs in both CG and CHG contexts were primarily located within TEs, more than 86 and 92% for CG and CHG, respectively (Fig. 4b). Considering that 85% of the maize genome is considered to be composed of TEs, this indicates that DMRs in the CHG context were preferentially targeting TEs. Such preference for DMRs in TEs was not apparent in the CG context. Relatively few DMRs were spanning both promoter and genes, with only 1–2% in both contexts. DMRs located in promoters were also relatively rare, with 2–4% depending on the context. DMRs in genes amounted up to 9 and 2% in CG and CHG contexts, respectively.

### Correlation between differential DNA methylation and transcriptional changes

**Fig. 5:**
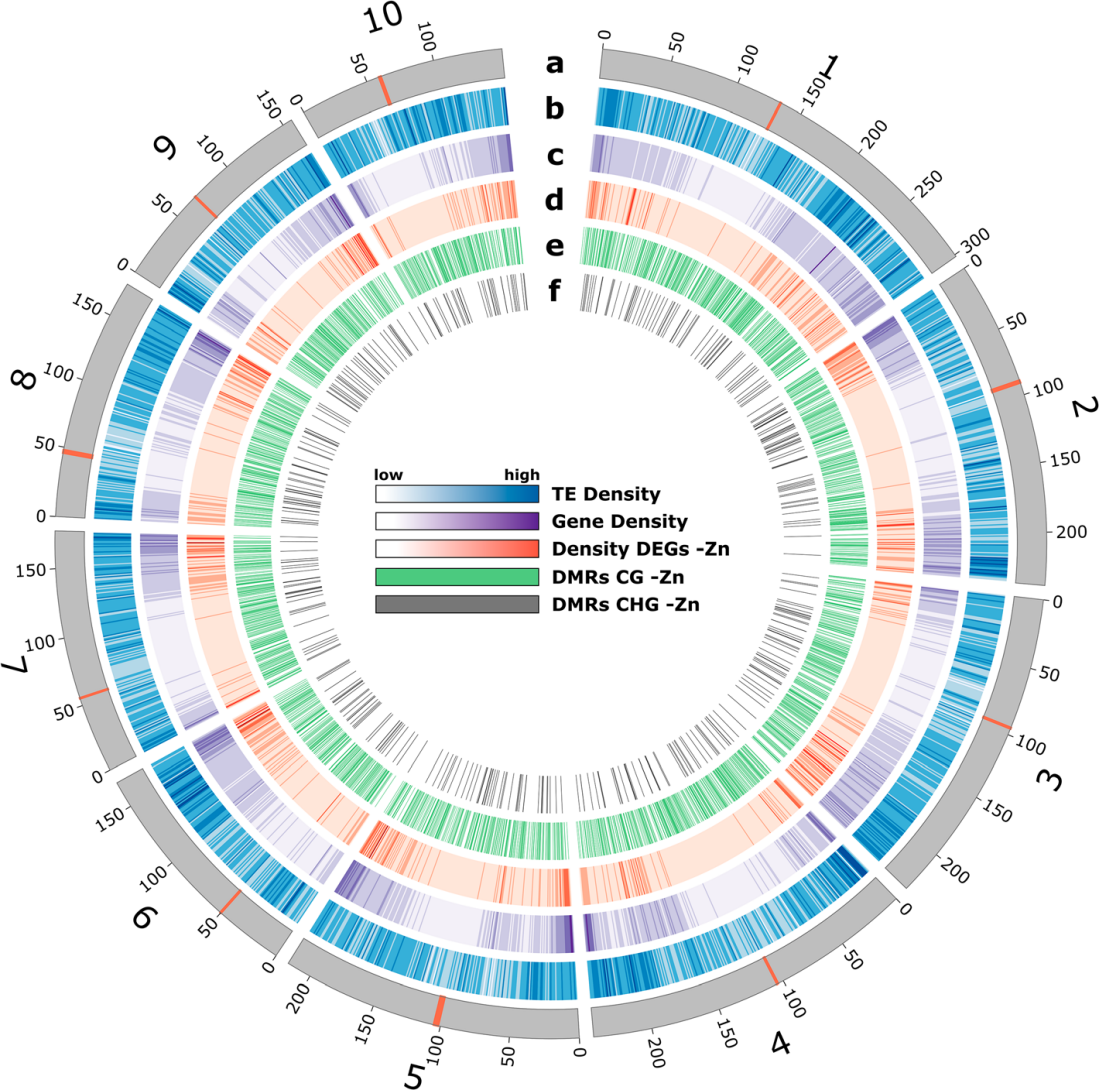
Distribution of different features across all 10 chromosomes of the B73 genome **(a)** Chromosomes (grey) with their centromere shown as red band. **b** Density of transposable elements (TEs, blue). **c** Gene density (violet). **d** Density of DEGs in -Zn (red). **e** Distribution of CG DMRs in -Zn (green). **f** Distribution of CHG DMRs in -Zn (dark grey)

The RRBS and RNA-sequencing data were then analyzed for possible correlations between methylation and gene expression differences. The distribution of TEs, genes, DEGs and DMRs in the entire ten chromosomes of the maize genome is shown in Fig. 5. Centromers [23] are marked with a red line. The enrichment of genes towards the terminal ends of each chromosome arm was associated with more DEGs in -Zn. CG and CHG DMRs, by contrast, were rather uniformly distributed across each chromosome and were preferentially localized in TEs.

**Fig. 6:**
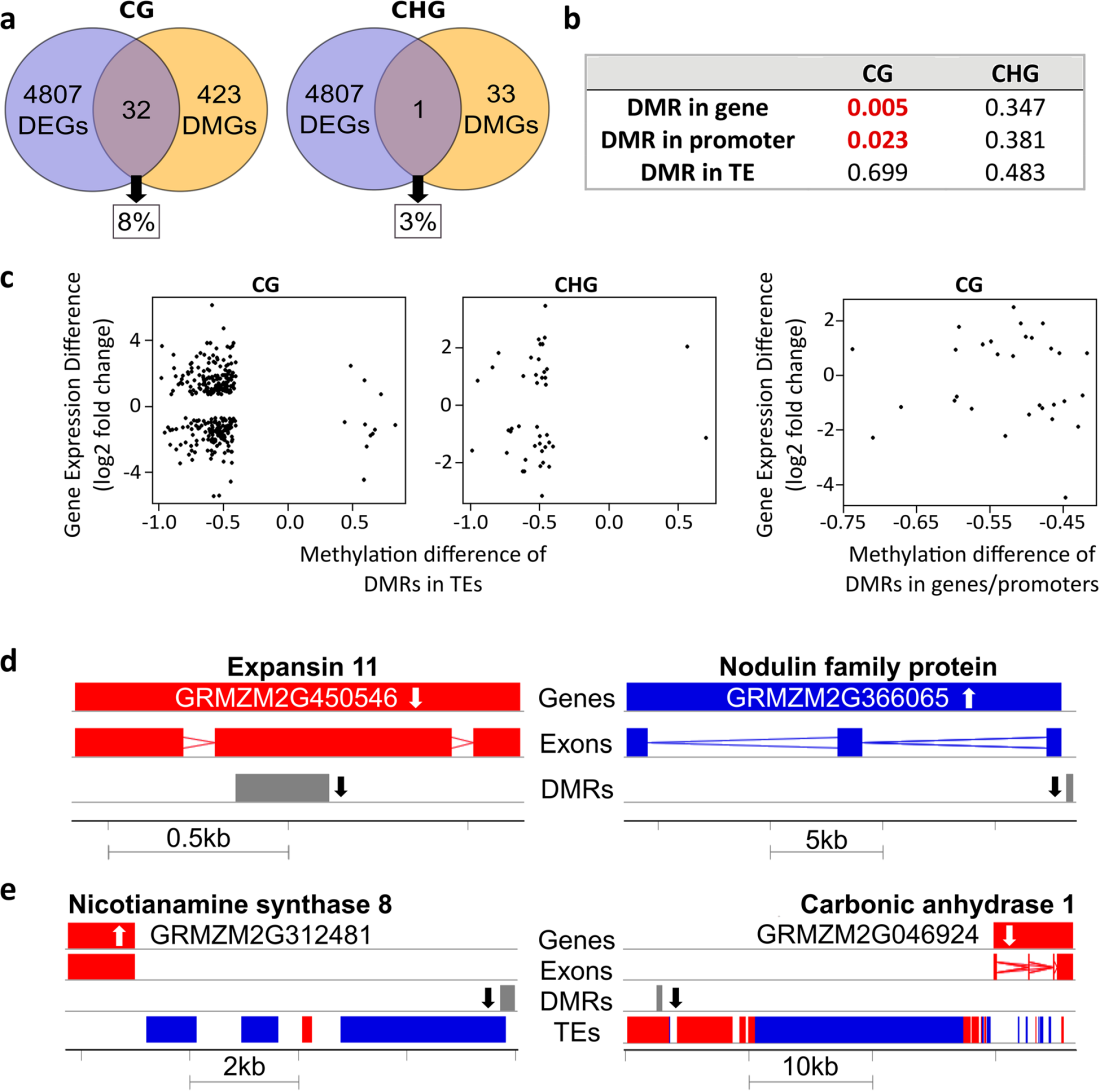
Relationship between gene expression and differential DNA methylation **(a)** Overlap between differentially expressed genes (DEGs) and differentially methylated genes (DMGs) in CG context (left) and CHG context (right). 8% or 3% of DMGs were also differentially expressed, respectively. **b** *P* values of Fisher’s exact test for correlations between DMRs in genes, promoters and neighboring TEs. **c** Scatterplots of differentially methylated genes that were also differentially expressed. Left: DMRs in adjacent TEs in CG context; middle: DMRs in adjacent TEs in CHG context; right: DMRs in promoters or gene bodies. **d, e** Examples for differentially methylated genes in CG context with differential expression. White arrow = gene expression up/down, black arrow = methylation up/down, red = forward strand, blue = reverse strand **(d)** DMRs in gene bodies/promoters of differentially expressed genes. **e** DMRs in TEs nearby differentially expressed genes

Since methylation was measured only in a representative fraction of the genome, but RNA-seq covered all differentially expressed genes, we calculated the percentage of differentially expressed genes containing a DMR in their gene body. The fraction of differentially expressed genes that had DMRs in the 2 kb region upstream of the gene (putative promoter) was also determined, as well as nearby downstream DMRs (Fig. 6a). In the CG context, 32 of the 423 differentially methylated genes covered by the RRBS were also differentially expressed, amounting for 8% of the genes with DMRs. In the CHG context, only a single gene was differentially methylated and differentially expressed at the same time, comprising only 3%.

To examine the statistical significance of the above observations, we considered only genes that were covered by at least 500 base pairs in the reduced representation methylome. By contrast, no length restriction was applied to differentially methylated TEs for testing the influence on the expression of the closest gene, because many TEs were very short and these were otherwise lost from the analysis. For the CG context, a significant correlation (at 5% level) of gene expression and the methylation in their promoters or genes themselves was suggested, with *p*-values of 0.023 and 0.005, respectively (Fig. 6b). On the other hand, there was no significant correlation between differentially methylated TEs and differential expression of the closest gene.

To further examine the relationship between differential methylation and gene expression, scatter plots were applied for differentially methylated TEs whose closest gene was differentially expressed, as well as for differentially expressed genes, which had a DMR in their promoter or gene body (Fig. 6c). The data points are skewed to the left part of the diagram, as methylation was predominantly reduced. Interestingly, differentially methylated promoters/gene bodies and differentially methylated TEs were both in up- or down-regulated genes, without preference for either change. Thus, even though there were significant correlations found for differentially expressed genes/promoters and the gene’s expression, gene expression can be repressed or stimulated by demethylation. A similar result was found if all genes were considered for the scatterplots (instead of only significantly differentially expressed ones, Additional file 1: Figure S2).

Among all genes that were differentially expressed and possessed a DMR in their gene body, in their 2 kb promoter or nearby transposable element, there were several genes that might be causally involved in the response to Zn deficiency. The position of the DMRs in the gene structure is shown for some differentially expressed gene examples in Fig. 6d. Among the differentially expressed genes discussed in Table 1, expansin genes were prominent and down-regulated under -Zn. A hypomethylation DMR was located in the gene body of expansin11, while a nodulin gene was up-regulated and hypomethylated just upstream of the transcriptional start site. Two examples for differentially methylated TEs near to differentially expressed genes are given in Fig. 6e. Nicotianamine synthase *ZmNAS8* was up-regulated and a carbonic anhydrase down-regulated in expression, while each of them was located near a hypomethylated TE. This illustrates that although hypomethylation was dominant, both up- and down-regulation of gene expression occurred upon hypomethylation.

As small RNAs play roles in the RNA -directed DNA methylation pathway, which is especially important for the CHH methylation, but also for CHG and CG contexts, we speculated that small RNAs might be decreased in the -Zn samples that had massively lost methylation. This is supported by the observation that -Zn samples contained a much lower ratio of small RNAs to total RNA, which amounted to 1.4%, compared to 9.7% in the controls (Additional file 1: Figure S3).

## Discussion

The prolonged growth of plants in Zn deficiency caused a range of changes in the roots, including transcriptomic as well as epigenetic changes. The Zn level in deficient plants (6.9 ppm) was well below the concentration typically considered as sufficient for maize (20 ppm). These stunted plants had similar iron concentrations, but they were slightly reduced in N and P, probably reflecting secondary metabolic effects from the prolonged Zn deficiency, but both macroelements were in the sufficiency range. Our further focus was on a representative fraction of the root methylome and its entire transcriptome.

### The maize root transcriptome upon prolonged Zn deficiency

Substantial expression of high affinity transporters for a certain nutrient is a key feature of typical prolonged deficiency, to provide efficient uptake of the small remaining traces of that nutrient. For Zn deficient roots, substantial up-regulation of zinc transmembrane transporter genes, which were previously found to be only marginally up-regulated by Zn deficiency in maize shoots and roots [9], was observed. These include *ZmZIP3,4,5,7,8* and a *ZIP1*-related gene, which were expressed under –Zn at similar elevated levels, but also nicotianamine synthase (*NAS*), which is involved in synthesizing the heavy metal chelator nicotianamine [7]. The most prominent gene from this gene family in –Zn was *ZmNAS5*, while carbonic anhydrase genes were slightly down-regulated in the -Zn maize roots, consistent with spinach and rice plants [24, 25].

Prolonged nutrient deficiency in roots may create secondary stress, but despite thousands of genes were differentially regulated, the -Zn transcriptome was consistent with much of the physiological knowledge of Zn deficiency. The up-regulation of purple acid phosphatase genes under -Zn in maize was also found in wheat [20]. Similar to results in Arabidopsis [7], some nodulin-like protein coding genes were down-regulated in -Zn. However, here some (uncharacterized) nodulin family genes were also highly up-regulated in the -Zn maize roots. Similar to Zn-deficient *Arabidopsis* roots, expansin genes were profoundly down-regulated [7], in agreement with smaller roots. Consistent with the literature is also the strong gene expression loss of superoxide dismutase, peroxidase and other enzyme genes needed for detoxification of reactive oxygen species [3]. Elevated hydrogen peroxide concentrations and insufficient superoxide dismutase activity are ameliorated by Zn addition in maize roots [26], but it remains unclear why this disturbed reactive oxygen detoxification under -Zn is not compensated by gene up-regulation of other detoxification enzymes that do not require Zn. In several species, the inability to maintain copper/zinc-dependent superoxide dismutase activity under Zn deficiency was not compensated by an up-regulation of other reactive oxygen pathways [3], which results in increased superoxide and hydrogen peroxide concentrations in the root tissue. Interestingly, it is well-established that Zn-deficient plants accumulate carbohydrates in their shoots, especially in the phloem sap of source leaves, but it was unclear whether this resulted from impaired phloem export of sucrose or decreased sink demand [3]. The finding that *SWEET13* sucrose channel genes were exceptionally up-regulated in roots may indicate that the plants invest heavily in sucrose efflux in roots. These efflux channels may then also be involved in the increased root sugar exudation typically found under Zn deficiency [3].

We also noted a tendency for reduced expression of genes involved in methylation maintenance. The transcriptional response to prolonged Zn deficiency affected many more genes than in *Arabidopsis,* but key components of the -Zn deficiency response were highly similar [7, 27].

### Deficiency-adapted methylome

Starting with the observation that methylation maintenance genes were suppressed under -Zn, we asked whether methylation was affected by -Zn. Previous analysis in *Arabidopsis* indicated methylation differences upon -Zn in roots and a minor methylation loss [27].

In contrast to *Arabidopsis*, a very strong loss in methylation frequency, especially in the CG and CHG contexts, was encountered in maize. This reduction in overall methylation, especially in the CG context, went along with DMRs being mostly hypomethylated. 97% of CG DMRs and 93% of CHG DMRs had lower methylation in -Zn than in the control. Slight visual symptom differences among individual shoots reflected slight variable stress levels in individual plants, which was mirrored by some variation within the (pooled) replicates. The adaptation of the methylation may be directly related to the lack of Zn or rather linked to a general stress response. In rice under phosphate starvation, DMRs were preferentially associated with starvation associated genes and TEs in their vicinity [13]. The occurrence of typical Zn-starvation-regulated differential gene expression hints at a Zn-specific adaptation in maize. Additionally, methylation was significantly related to expression of some of the differentially expressed genes.

The de novo more flexible CHH methylation is commonly thought to respond faster to environmental conditions than the symmetric CHG and CG contexts, but to our surprise, the CG context was most affected, while the CHH context was barely methylated and only a negligible number of CHH DMRs were found. This is in contrast to phosphate-starved rice [13], even with loosened criteria for defining DMRs. Li et al., 2015 also recognized low methylation in the CHH context in maize and only very few DMRs in five different maize inbred lines, including B73, which was studied here. The same discovery has been made by Eichten et al., 2013 who also found very low (< 10%) methylation in the CHH context of the inbred lines B73 and Mo17. In *Arabidopsis thaliana* a similar low methylation level (1.7%) in the CHH context was found (Law and Jacobsen, 2011), similar to the plants in our experiments (1.3% in control plants). DNA methylation differences were recorded after 4 weeks in -Zn, but changes might occur within days, as in phosphate-starved rice [13]. A time course of methylation changes during development and treatments would be informative to better understand the relationship of these changes.

### Interaction between DNA methylation and gene expression

Genome wide DNA methylation changes upon nutrient-stresses and the correlations with gene expression have rarely been studied in other plants than rice or *Arabidopsis thaliana*. It is clear that possibly harmful activities of transposable elements are silenced by hypermethylation of transposons (Dowen et al., 2012; Slotkin and Martienssen, 2007; Tan, 2010; Tsaftaris et al., 2003; Yong-Villalobos et al., 2015). Methylation changes in transposons frequently affect the expression of adjacent genes (Lisch and Bennetzen, 2011; Mirouze and Vitte, 2014; Slotkin and Martienssen, 2007). These mechanisms are probably of less importance in *Arabidopsis*, as this plant has few transposons and low methylation. In maize with its 2.3 Gb giant genome that is full of TEs, genes are spread over large distances, as are gene regulatory sequences (Schnable et al., 2009; Tenaillon et al., 2011). Parallel representative bisulfate DNA sequencing and RNA-seq identified correlated changes in the methylome and transcriptome of maize roots and revealed a strong decrease in methylation. The percentage of up- and down-regulation of zinc deficiency-regulated DEGs was almost the same (~ 50% each), which led to a significant repression of many gene categories in -Zn, including reactive oxygen handling and cell wall categories. We could show that several of the genes containing a DMR in their promoter or gene body were associated with altered gene expression, in contrast to the situation in P and N deficiencies, where no clear correlation was found [22]. However, differential methylation in transposable elements had a significant effect on the expression of nearby genes in P and N deficiency, while this was not found for Zn deficiency. However, due to the large maize genome and the very long stretches of genomic DNA that may be involved in regulating genes in maize, we may have missed correlations with more distant regions. In rice, by contrast, hypermethylated TEs were often close to induced genes [13], while in *Arabidopsis* hypermethylation of TEs frequently leads to decreased expression of nearby genes [28–30]. In -Zn maize roots, most TEs were hypomethylated, but a similar number of closeby genes was up- or down-regulated. The same was observed for demethylated genes or promoters in -Zn, their expression was inconsistently increased or decreased. Thus, the potential general role of hypermethylation on gene expression remains unclear. Rather, literature as well as our results suggest strong species- and nutrient-specific methylation and transcription adaptation. That methylation level in promoters and gene bodies is correlated with gene expression is further supported by our data, but it is impossible to deduce causality from these data.

Even though hypomethylation is typically accompanied by an increased expression of transposable elements, this was not supported by our dataset, although we need to note that TE expression was not explicitly focused on. While under nitrogen and phosphorus deficiencies the overall expression of TEs was at least moderately increased [22], their expression in -Zn was even slightly reduced (by 9%).

Small RNAs are associated with DNA methylation especially in the CHH context (which was little methylated) and we noted a tremendous reduction in small RNA amount in -Zn samples. This may suggest further cross-talk between the different methylation pathways and implicates that loss of methylation under -Zn in maize comes along with a reduction in small RNA amount even though there was not much change encountered in the expression of enzymes involved in the RdDM pathway. However, the quantification and reduction in small RNAs using the BioAnalyzer has also to be taken with caution, as small RNA levels may be subject to artifacts such as RNA degradation and inaccurate length estimation.

### Methylation, other stresses and potential function

Usually, genomic instability is attributed to DNA hypomethylation in plants, humans, animals and fungi [31–33]. It was also frequently assumed that adaptation to stress is brought about by increased genomic flexibility and instability as well as increased transposon activity [31, 34–36]. As our results show a massive loss of DNA methylation in maize roots due to Zn and nitrogen deficiency in maize [22], increased chromosome rearrangements might result from demethylation, beside direct regulation of gene expression. Only limited correlation between DNA methylation and gene expression is now frequently found [37] and conclusions about these correlations are often contradictory, which solidifies the assumption of an additional function of DNA hypomethylation.

The functions of DNA methylation in somatic tissues, such as roots or leaves, may be quite distinct from those in the germline, where DNA methylation as an epigenetic mechanism may contribute to transgenerational inheritance. The level of DNA methylation is important for regulation of TE activity, the frequency of recombination events as well as the occurrence of mutations [31, 34, 38]. As suggested previously for other stresses, controlled increase in chromosome rearrangements as well as increased activity of transposable elements might enhance adaptational processes to -Zn conditions [31, 34].

Not only genome-wide hypomethylation, but also local loss of methylation was found to increase rearrangement of genes. The hypomethylated genes were found to be resistance gene-like loci [39]. This hints at the ability of plants to highly control the occurrence of events caused by genomic instability for ‘controlled’ increase of genetic diversity, while balancing the associated risks coming along with genomic instability [34]. The strong global loss of DNA methylation and a particularly strong loss at the centromere and pericentromeric regions may be relevant in this context, as the activity of the TEs as well as crossovers at the centromeric region are usually suppressed by DNA methylation [40]. To avoid adverse effects of excess genome instability caused by hypomethylation (especially at the centromere, where crossovers might lead to chromosome breakage or loss [41]), also epigenetic factors other than DNA methylation might exert control to balance genomic instability. Thereby, global hypomethylation provides the ability for fast gene expression regulation and plasticity of the plants as well as controlled increases of genomic instability at certain locations. This assumption is substantiated by no overall increase of recombination frequency [40], but a global redistribution of these events due to hypomethylation, indicating interaction of different (epigenetic) mechanisms. It was even suggested that plants might be able to ‘recruit’ retrotransposons under stress conditions to foster evolution [42]. Under nitrogen and phosphorus deficiency in maize there was indeed an increase in the overall expression of TEs found [22]. The slight decrease in TE expression under -Zn might be due to the strong intensity of the stress or indicating stress-specific mechanisms.

Furthermore, plants exposed to ionizing irradiation stress were hypermethylated [35]. The authors suspected that hypermethylation in these plants prevents excess genomic instability induced by radiation but still propose mutations and recombination as contributing mechanism by which plants adapted to the radiation stress. Similarly, a general hypermethylation in progeny of virus-infected tobacco plants with local hypomethylation increased frequency of recombination events [34]. Hypermethylation was also found in tobacco due to osmotic stress [38]. However, cold stress induced hypomethylation in maize roots [43] and heavy metal stress was shown to cause hypomethylation in hemp and clover [44].

Though there is only limited evidence for the heritability of changes in genome stability due to DNA methylation changes, some researchers reported increased recombination in at least the direct progeny of stress-exposed plants [34, 35, 39]. Additionally, changes in genome stability can not only be inherited meiotically, but also mitotically [34]**.** Thereby it can enhance genetic diversity by vegetative reproduction, e.g. by somatic recombination events [45], providing the chance for increased adaptation and plasticity especially on the population level in plants.

To verify the conclusion that the strong loss of DNA methylation not only influences gene expression but also plays a role in fostering genetic diversity by controlled increase in recombination and mutation frequencies, further experiments will be necessary.

DNA methylation loss might function in increasing overall plasticity and reactivity of plants under stress, as well as increasing genomic instability. Other epigenetic mechanisms, like histone modifications, might balance the effects of methylation loss by inhibiting excess chromosome rearrangements. Therefore, simultaneous investigation of DNA methylation as well as histone modifications might give a clearer picture of the different factors influencing gene expression regulation and adaptation strategies and the fine-tuning of genomic instability.

Another important aspect to be tested is the heritability of such adaptations. To consider meiotic heritability, generative cells, like pollen, should be investigated for methylome changes and frequency of recombination events as well as tissues of the F1 generation of stressed parents (e.g. endosperm and embryo). Mitotic heritability could be tested in plants that frequently propagate through vegetative reproduction.

The importance of increased genomic instability as evolutionary driver could be especially important on the population level. Hereby, the genetic diversity in (wild) plants grown on deficiency soils or on fully supplied soils could be compared by looking for differences in methylome and transcriptome data and investigating if the stressed population shows new traits or differences in genes important for stress response. Furthermore, the influence of stress-type, stress-intensity and species on recombination events, double-strand breaks or transposable element activity should be tested.

## Conclusions

This work substantiates that DNA methylation changes in roots under nutrient deficiency. This is associated with gene expression changes that are related in nutrient-, species- and potentially organ-specific ways. How this affects the plasticity of the plants and functions as evolutionary driver by speeding up the development of new, potentially beneficial traits, should be tested in more detail in the future. The fact that different nutrient deficiencies (including macro- and micronutrients) resulted in methylation loss (though to a different extent) in maize roots, stimulates ideas for a range of further experiments. These could not only promote understanding of population genomics and aid evolutionary studies. As recombination is important to introduce new desired traits into plants during breeding, knowledge about plant-intern mechanisms altering recombination and TE activity through methylation changes could be artificially induced during breeding, enhancing velocity of the breeding processes, but it is required that the methylation in the germline responds similarly as somatic tissues to such stimuli [46, 47].

## Materials and methods

### Plant growth and sampling

Seeds of the inbred line B73 of *Zea mays* (from Dr. Schipprack of the department of Plant Breeding, University of Hohenheim) were first surface-sterilized with a 10% H_2_O_2_ solution by rinsing them for 2 min and removing H_2_O_2_ by washing the seeds in distilled water. For 24 h, the seeds were incubated in a 10 mM CaSO_4_ solution and were then put between foam sheets soaked in 3 mM CaSO_4_ solution for 4 days for germination. Afterwards, they were set into 2.8 L of a nutrient solution containing 0.1 mM K_2_SO_4_, 0.12 mM MgCl_2_, 0.5 mM Ca(NO_3_)_2_ and 20 μM KH_2_PO_4_, 0.2 μM H_3_BO_3_, 0.1 μM MnSO_4_, 0.1 μM ZnSO_4_, 0.04 μM CuSO_4_ and 2 nM (NH_4_)_6_Mo_7_O_24_. After 3 days, control plants were put on fully supplied maize hydroponic solution (2.8 l) and -Zn plants on a modified, Zn-deficient solution. The solution for control plants contained 0.5 mM K_2_SO_4_, 0.6 mM MgCl_2_, 2.5 mM Ca(NO_3_)_2_ and 0.1 mM KH_2_PO_4_. The latter was raised to 0.2 mM in the 3rd treatment week and to 0.5 mM in week 4. Furthermore, 1 μM H_3_BO_3_, 0.5 μM MnSO_4_, 0.5 μM ZnSO_4_, 0.2 μM CuSO_4_, 0.01 μM (NH_4_)_6_Mo_7_O_24_ and 100 μM Fe-Sequestrene were added. This was raised to 200 μM during the first nutrient solution change and from the second solution change on it stayed at 300 μM. Zn deficient plants were treated the same, except for the following modifications: The amount of KH_2_PO_4_ was not raised, but kept at 0.1 mM. Instead of Fe-Sequestrene, 300 μM of Fe-EDTA were added to the solution, in case that Fe-Sequestrene contained traces of Zn. Zn was omitted from the solution, but a residual Zn in the sub-μM range was always detected in the nutrient solution, probably resulting from the plastic containers in which the plants were grown. At week 3 and 4 of the treatment, 0.1 μM ZnSO_4_ were given to the solution for 24 h to prevent dying of the plants. Nutrient solution was exchanged once after one week and then every 3 days.

The plants grew in a climate chamber, enabling controlled growth conditions. A simulated day length of 16 h at 25 °C and a night length of 8 h at 20 °C was applied. Photosynthetically active photon flux density (PFD) was 400 μmol m^− 2^ s^− 1^ and humidity was kept at 60–80%. For each condition 6 plants were grown, with 2 plants per pot. After 5 weeks (corresponding to 4 weeks of treatment), root and shoot material was harvested, whereby the two plants in one pot were pooled, resulting in 3 replicates (with 2 plants per replicate) for both control and -Zn.

### Nutrient analysis

Leaf samples were ground to a fine powder before being digested in a microwave oven. Zinc, iron, phosphorus and nitrogen concentrations were measured. The latter three were measured to confirm nutrient specificity in -Zn samples. Zinc and iron were determined via inductively coupled plasma mass spectrometry (ICP-MS), while phosphorus was measured as orthophosphate after addition of molybdate-vanadate reagent via UV-VIS spectroscopy and nitrogen measurement was done after Kjeldahl [48].

### RNA sequencing on total RNA samples and transcriptome analysis

Total RNA was extracted from root material of control and -Zn plants via the analytikjena innuPREP Plant RNA Kit, according to the manufacturer’s manual. The quantity and quality of the RNA were checked with the Thermo Scientific Nanodrop 2000c Spectrophotometer and the Agilent 2100 Bioanalyzer. All RNA samples had a purity of OD260/280 ≥ 1.8. A Truseq 160 basepair (bp) short-insert library and 100 bp paired-end sequencing on Hiseq4000 were made at the Beijing Genomics Institute (BGI, China). The clean data were quality checked via the FastQC tool version 0.11.5 (by Babraham Bioinformatics) and reads were aligned to the *Zea mays* reference genome (AGPv3), provided by the maizeGDB (Maize Genetics and Genomics Database) [49], with the options --phred64, −-dta-cufflinks, −-no-mixed and --no-discordant. Annotation files AGPv3 were used [49]. The assembly was done with cufflinks from the cufflinks suite of tools version 2.2.1 [50] with default options and the --GTF-guide and --no-effective-length-correction. After merging the assemblies with cuffmerge (with -g and -s options), cuffdiff was used to find differentially expressed genes (DEGs) with --compatible-hits-norm, −b, −u and otherwise default options. Transcript amounts are given as fragments per kilobase of transcript per million mapped reads (FPKM). Differences between the conditions are given as log_2_-fold changes as log_2_(FPKM_-Zn_/FPKM_Control_). The threshold for differentially expressed genes (after Benjamini-Hochberg correction for multiple testing) was set to *p* < 0.05. Overrepresentation of differentially expressed gene categories was determined by a Parametric Analysis of Gene Set Enrichment (PAGE) using the reference genome *Zea mays* AGPv3.30 and the agriGO analysis toolkit version 1.2 [51]. Bonferroni multi-test adjusted values with different expression (p < 0.05) were used.

### Reduced representation bisulfite sequencing of DNA samples and methylome analysis

After harvest of the maize roots, the material was ground to a fine powder and the DNA was extracted via Qiagen DNeasy Plant Mini Kit according to the manual. DNA samples were checked for quantity and quality via Thermo Scientific Nanodrop 2000c Spectrophotometer and Qubit Fluorometric Quantitation. Only samples with an OD260/280 bigger or equal to 1.8 were used for further processing. The DNA was digested with MspI followed by selection of fragments between 40 to 220 bp. These fragments were used to construct a reduced representation bisulfite sequencing library. 100 bp paired-end sequencing on Illumina Hiseq2000 was done at Beijing Genomics Institute (BGI, China). A quality checks of the clean data were done with FastQC version 0.11.5 (Babraham Bioinformatics). To increase the quality further, FastX-Toolkit version 0.0.13 by Hannon Lab was used to cut off the first 4 and 6 last bp of all reads. Mapping was done via BS-Seeker2 version 2.0.10 [52] using the *Zea mays* reference genome AGPv3. BS-Seeker2 virtually cuts this genome with MspI and size-selects sequences of desired length. Parameters used were -l 20, −u 400, −-aligner = bowtie2 and otherwise default options. By virtually digesting the reference maize genome with MspI and subsequent size-selection of DNA fragments between 40 and 220 bp length, the theoretically covered genome fraction (14%) was determined. A broader range of size-selection (namely 20–400 bp length) than that for the digested DNA samples was used for the virtual reduced representation genome to account for inaccuracies during size-selection of the digested DNA samples. The bowtie2 as short read mapper and default settings were used for alignment and methylation calls [53]. DMRcaller version 1.2.0 [54] in R version 3.2.4 was used to determine differentially methylated regions (DMRs) by pooling the methylation level information from all three replicates. Smoothing was done via noise_filter with triangular kernel [55] for computing differentially methylated cytosines. DMRs were characterized between 50 and 500 bp, containing at least 4 cytosines (with at least 4 reads per cytosine), resulting in a methylation difference of at least 40% in comparison to the control and a *p*-value < 0.01. DMRs located within genes were determined with gene information taken from maize annotation files in AGPv3 [49]. The region comprising 2000 bp upstream of a gene was defined as a gene promoter. Transposable elements (TEs) annotation from the Unité de Recherche Génomique Info [56] was used.

### Correlating transcriptome data with methylation information

Correlations between differential methylation in genes, promoters or TEs with gene expression were investigated by Fisher’s exact test on a 2 × 2 contingency table in R version 3.3.3. Only genes covered by at least 500 bp by the reduced representation genome were taken into account for quantification of differentially methylated genes, promoters and their gene expression, to avoid false negative results. More distant (the closest) TEs were taken into account if they were covered by the reduced representation genome to avoid loss of very short transposable elements. BEDTools version 2.26.0 [57] was used to determine the closest gene to each transposon, regardless of being upstream or downstream.

### Quantification of small RNAs

Small RNA was extracted from the same root material that was used for total RNA and DNA extractions. The extraction was done according to the analytikjena innuPREP Micro RNA Kit Manual. The samples were tested in the Thermo Scientific Nanodrop 2000c Spectrophotometer for purity and only samples with OD260/280 ≥ 2.0 were used. The amount of small RNAs was determined in the Agilent 2100 Bioanalyzer according to the Agilent Small RNA Kit Guide by Agilent Technologies. The ratio between the amount of sRNAs (15–30 nt length) and the total amount of RNA was determined. As 21–24 nt sRNAs are involved in the RdDM pathway, the 15–30 nt sRNA range was chosen to make sure that all 21–24 nt sRNAs were taken into account. Significant changes in the amount of sRNAs were determined via one-way ANOVA in R version 3.1.1.

## Additional file

Additional file 1: **Table S1.** Alignment rate of RNA-Sequencing. Alignment rates were lower in -Zn. Values are averaged among replicates. **Figure S1.** Cytosine coverage in CG, CHG and CHH contexts. Higher cytosine coverage in each context is observed under -Zn. **Table S2.** Alignment output of RRBS libraries. Similar mappability in control and -Zn of RRBS libraries. Values are averaged among replicates and shown in millions.

## Abbreviations

DEG: differentially expressed gene
DMR: differentially methylated region
RdDM: RNA dependent DNA methylation
RRBS: reduce representation bisulfate sequencing
TE: transposable element
Zn: zinc
