# Supplementary material for: The transcriptome of zinc deficient maize roots and its relationship to DNA methylation loss
Source: BMC Plant Biol. 2018 Dec 27;18:372. doi: 10.1186/s12870-018-1603-z (PMC6307195; doi:10.1186/s12870-018-1603-z)

Supplementary material for “The transcriptome of zinc deficient maize roots and its relationship to DNA methylation loss” by Svenja Mager, Brigitte Schönberger and Uwe Ludewig

**Additional file 1: Table S1 Alignment rate of RNA-Sequencing.**

Values are averaged among replicates.

|  | **Control** | **-Zn** |
| --- | --- | --- |
| Paired reads | 28.0 M | 27.7 M |
| Unaligned reads | 2.3 M | 5.7 M |
| Multiply aligned reads | 2.7 M | 2.5 M |
| Uniquely aligned reads | 23.0 M | 19.5 M |
| **Alignment rate** | **91.8 %** | **79.6 %** |

**Additional file 1: Figure S1 Cytosine coverage in CG, CHG and CHH contexts.**


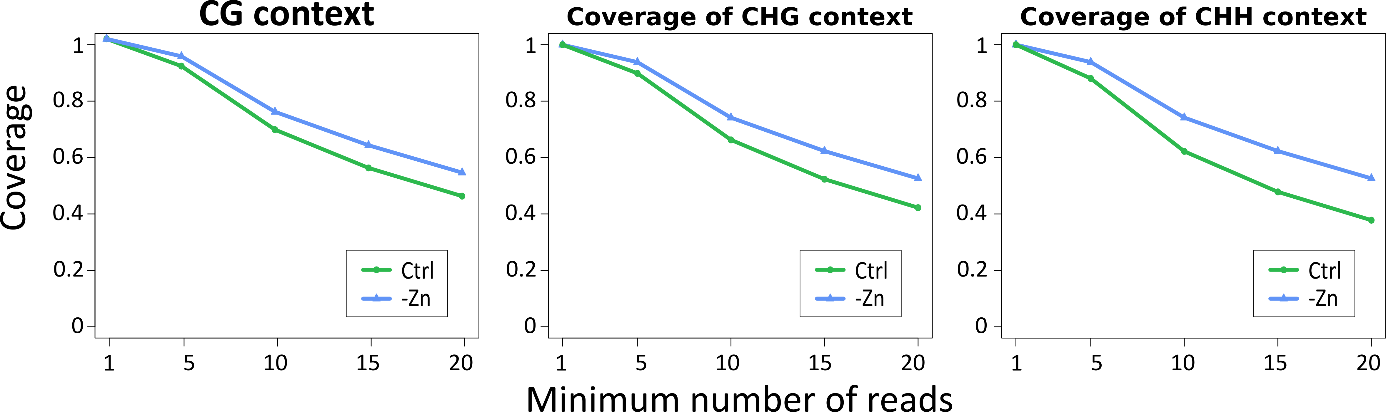


**Additional file 1: Table S2 Alignment output of RRBS libraries.**

Values are averaged among replicates and shown in millions.

|  | **Control** | **-Zn** |
| --- | --- | --- |
| Raw BS-read pairs | 35.40 | 42.86 |
| Multiple hits reads | 0.13 | 0.13 |
| Unmapped read pairs | 18.20 | 21.78 |
| Uniquely aligned read pairs | 17.23 | 21.08 |
| **Mappability** | **48.62%** | **49.16%** |

**Additional file 1: Figure S2 Scatterplots of genes with differential methylation in gene/promoter or adjacent TE and their expression.** Left: Genes with differential methylation in gene or promoter Right: Genes close to a DMR in an adjacent TE.


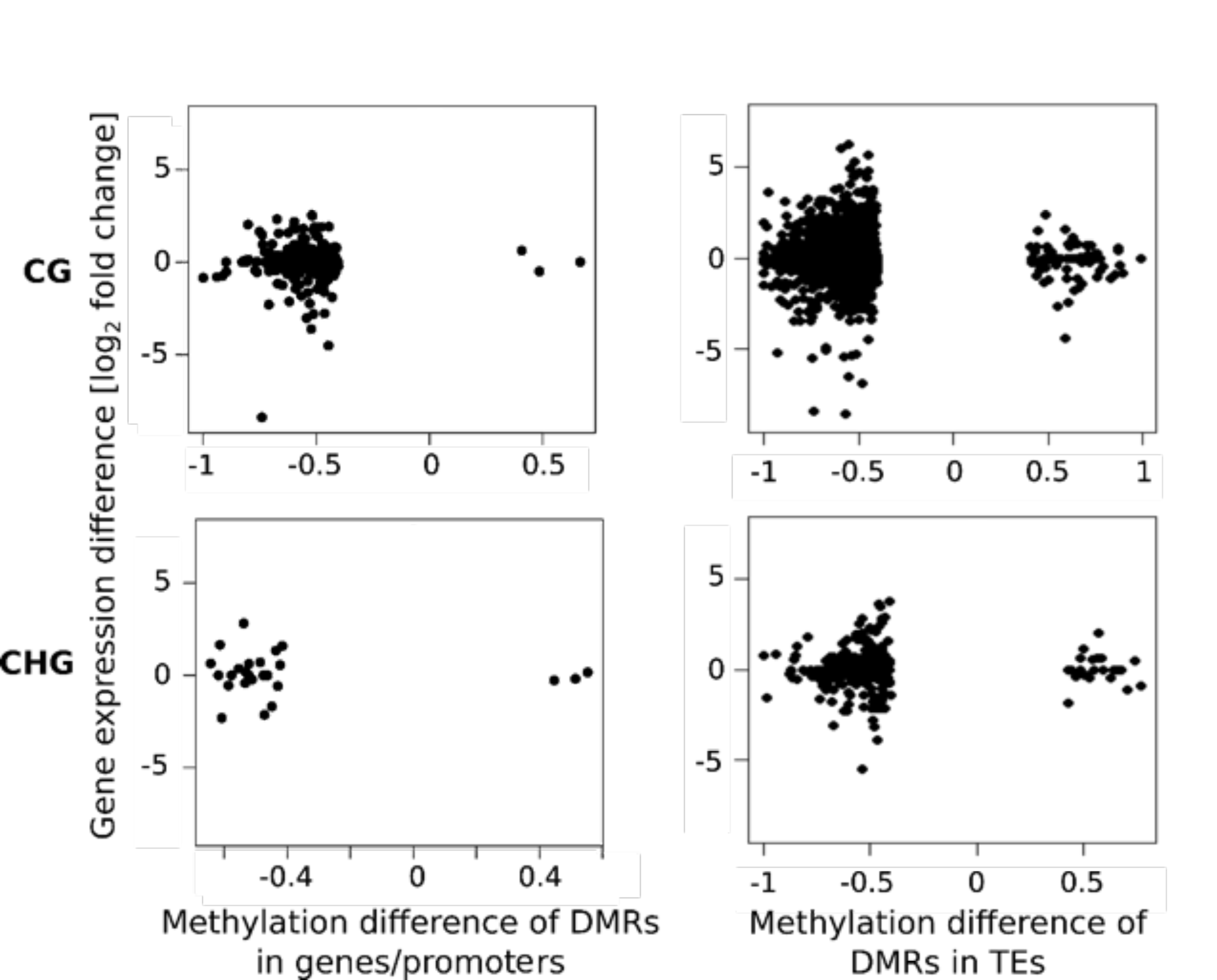


**Additional file 1: Figure S3 Relative amount of small RNAs** Small RNA fraction of total RNA amount.


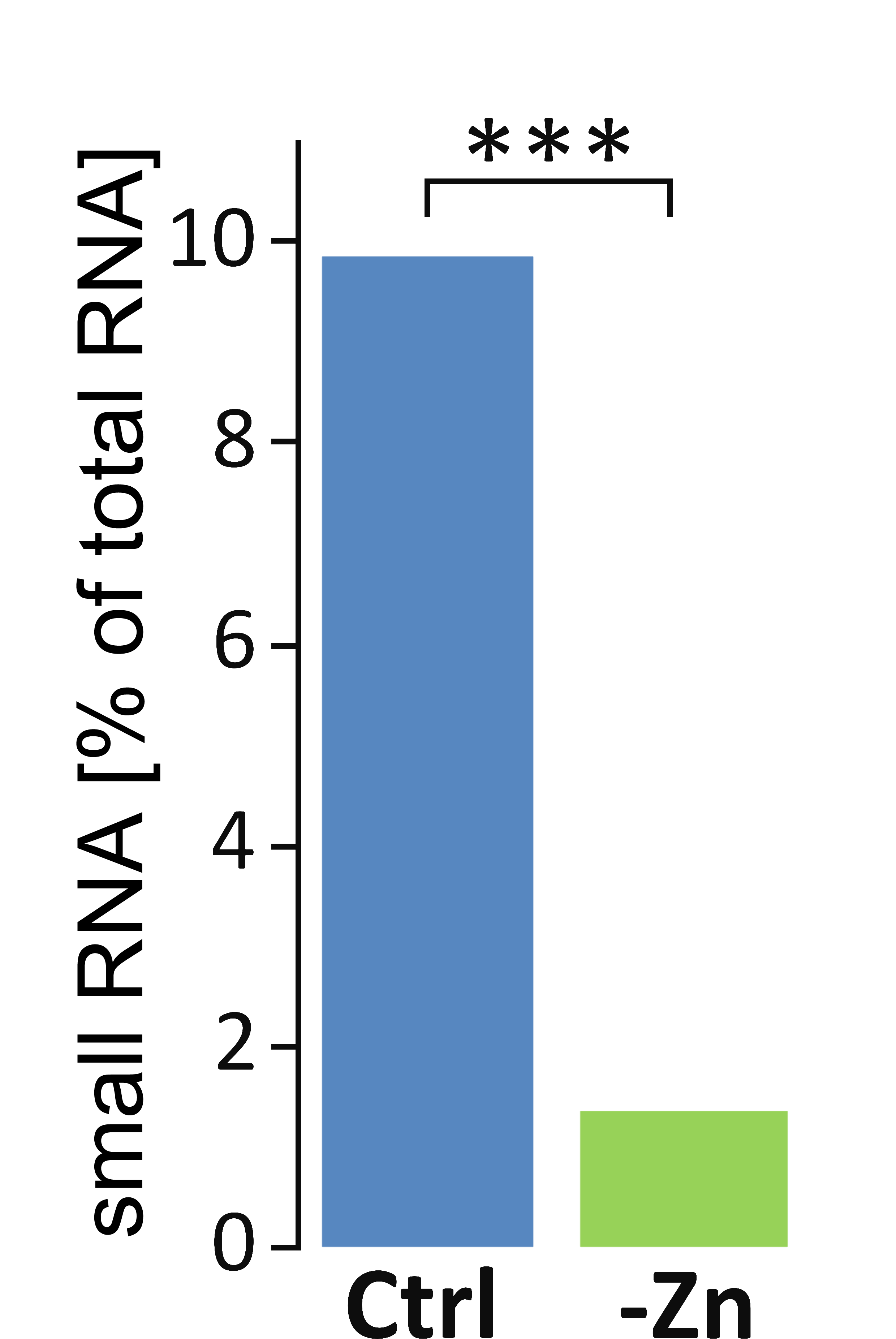

Supplement: Supplementary file 1 — Table S1. Alignment rate of RNA-Sequencing. Alignment rates were lower in -Zn. Values are averaged among replicates. Figure S1. Cytosine coverage in CG, CHG and CHH contexts. Higher cytosine coverage in each context is observed under -Zn. Table S2. Alignment output of RRBS libraries. Similar mappability in control and -Zn of RRBS libraries. Values are averaged among replicates and shown in millions. [file 12870_2018_1603_MOESM1_ESM.docx]
